# Remodeling of host phosphatidylcholine by *Chlamydia* acyltransferase is regulated by acyl-CoA binding protein ACBD6 associated with lipid droplets

**DOI:** 10.1002/mbo3.234

**Published:** 2015-01-21

**Authors:** Eric Soupene, Derek Wang, Frans A Kuypers

**Affiliations:** Children's Hospital Oakland Research InstituteOakland, California USA

**Keywords:** Acyl-CoA binding protein, acyltransferase, lipid droplets, phosphatidylcholine

## Abstract

The bacterial human pathogen *Chlamydia trachomatis* invades cells as an infectious elementary body (EB). The EB is internalized into a vacuole that is hidden from the host defense mechanism, and is modified to sustain the development of the replicative reticulate body (RB). Inside this parasitophorous compartment, called the inclusion, the pathogen survives supported by an active exchange of nutrients and proteins with the host cell. We show that host lipids are scavenged and modified into bacterial-specific lipids by the action of a shared human-bacterial acylation mechanism. The bacterial acylating enzymes for the essential lipids 1-acyl-*sn*-glycerol 3-phosphate and 1-acyl-*sn*-phosphatidylcholine were identified as CT453 and CT775, respectively. Bacterial CT775 was found to be associated with lipid droplets (LDs). During the development of *C. trachomatis*, the human acyl-CoA carrier hACBD6 was recruited to cytosolic LDs and translocated into the inclusion. hACBD6 protein modulated the activity of CT775 in an acyl-CoA dependent fashion and sustained the activity of the bacterial acyltransferase by buffering the concentration of acyl-CoAs. We propose that disruption of the binding activity of the acyl-CoA carrier might represent a new drug-target to prevent growth of *C. trachomatis*.

## Introduction

Integrity of eukaryotic membranes is maintained by processes that require the continuous renewal of lipid molecules. Lipids can be replaced by newly synthesized molecules, or modified in the membrane by de-acylation and re-acylation processes also known as the Lands’ pathway (Lands 1960). Oxidized acyl chains and acyl chains of an inappropriate length or configuration, are removed by the action of phospholipase A_2_ (PLA_2_) (van den Berg et al. 1993; Burke and Dennis 2009). The re-acylation of the resulting lysophospholipid (lysoPL) is achieved by a co-enzyme A (CoA) and ATP-dependent two-step process (Lands 1960). Fatty acids are activated to acyl-CoAs by membrane-bound long-chain acyl-CoA synthetases (ACSL) and are then transferred to the lysoPL acceptor by membrane-bound acyl-CoA:Lysophospholipid-acyltransferases (LPLAT) (Lands 1960; Lands and Merkl 1963; Merkl and Lands 1963; Lands and Hart 1965). The combined de-acylation, activation and re-acylation represents the Lands’ pathway supporting lipid repair and remodeling of membranes, as well as the removal of bio-active and pro-inflammatory lysoPL molecules (Lands 1960).

The obligate intracellular bacteria *Chlamydia trachomatis* multiplies in the human host cells inside a newly formed parasitophorous vacuole, called an inclusion (Hackstadt et al. 1997). Confined and protected in the inclusion, the bacteria intercept nutrients and metabolites of the host cell to support its growth. Host-derived lipids, ceramide, sphingomyelin and glycerophospholipids, and lipid vesicles are scavenged to sustain the expansion of the inclusion membrane and the synthesis of the bacterial membrane (Hackstadt et al. 1995, 1996; Scidmore et al. 1996; van Ooij et al. 2000; Tse et al. 2005; Beatty 2006; Kumar et al. 2006; Cocchiaro et al. 2008; Derre et al. 2011; Elwell et al. 2011; Cox et al. 2012). Inhibition of the host lipid metabolism impaired bacterial growth and fusion of inclusions (Hackstadt et al. 1995, 1996; Kumar et al. 2006; Robertson et al. 2009; Cox et al. 2012). Of particular interest was the finding that *C. trachomatis* lacks de novo synthesis for phosphatidylcholine (PC) and depends on the host to provide 40% of total bacterial lipids (Wylie et al. 1997). However, bacterial phospholipid (PL) species contain branched-fatty acids that do not exist in eukaryotic lipids and host membranes (Wylie et al. 1997; Lim and Klauda 2011). The necessary conversion of the straight-chain containing PC lipids of the infected cells into bacterial specific moieties adds an additional step in the inclusion expansion that could be susceptible to inference and could limit the growth of the pathogen. This scavenging/remodeling process shares features with components of the host membranes Lands’ pathway and several lines of evidence indicated that this de-acylation/re-acylation cycle takes place after transit of host lipid molecules to the inclusion membrane. PC molecules fluorescently labeled at the sn-2 position were detected in the inclusion membrane but not in the bacterial membrane (Hackstadt et al. 1995, 1996), whereas bacterial-PC was radiolabeled when a precursor of branched-fatty acids synthesis, [^14^C]-isoleucine, was added to the culture medium (Wylie et al. 1997). These observations established the presence of a de-acylating enzyme cleaving the sn2-fluorescent chain and the presence of a re-acylating enzyme transferring the *Chlamydia*-synthesized branched-chains fatty acid onto the lysoPC acceptor. The de-acylating enzyme is a cytosolic PLA_2_, which is activated by phosphorylation and is recruited to the inclusion membrane upon stimulation of a MERK-ERK MAP kinase signaling cascade by *C. trachomatis* (Su et al. 2004; Du et al. 2011). We have identified that long-chain acyl-CoA synthetase 3, hACSL3, a membrane-bound protein of the mitochondria and Golgi apparatus (Obata et al. 2010), was recruited into the lumen of the *C. trachomatis* inclusions in human cells (Soupene et al. 2012). We determined that an ER-bound re-acylating enzyme for PC (hLPCAT1; acyl-CoA:lysophosphatidylcholine acyltransferase 1) (Nakanishi et al. 2006) was mobilized to the lipid network surrounding the inclusion membrane but was not present inside the inclusion (Soupene et al. 2012). Additional acyltransferase enzymes, from the host or the pathogen, are needed for the re-acylation of lysophospholipids to branched-chain lipids inside the inclusion.

We report the identification of several *C. trachomatis* enzymes implicated in the production of bacterial lipids. The lysophospholipid acyltransferase LPAAT enzyme CT453 is the *Chlamydia* homologue of *Escherichia coli* PlsC (Coleman 1990), which is essential for de novo synthesis of bacterial glycerophospholipids. CT775 is a lysophosphatidylcholine acyltransferase (LPCAT) enzyme that re-acylates host lysoPC into *Chlamydia-*specific PC molecules. We confirmed that lipid droplets (LDs) produced by the host cells were translocated into the inclusion (Cocchiaro et al. 2008), and determined that the bacterial LPCAT enzyme was associated with LDs. We show that a member of the human acyl-CoA binding protein family, hACBD6 (Soupene et al. 2008b), which is not associated with LDs in un-infected cells, binds to LDs during development of *C. trachomatis* and expansion of the inclusion; hACBD6 was exhaustively removed from the nucleus of the infected host cells and was translocated into the lumen of the inclusion apparently in association with LDs. In vitro, we determined that hACBD6 modulated the acyltransferase activity of CT775 and the formation of PC. hACBD3, a Golgi-bound acyl-CoA binding protein (Zhou et al. 2007) which is not translocated in the inclusion (Soupene et al. 2012), was less efficient in controlling acylation of lysoPC by the *Chlamydia* enzyme. Those results suggested that the association of host and bacterial proteins to LDs might facilitate their transfers across the inclusion membrane and that human proteins released in the lumen of the inclusion affect bacterial driven processes.

## Materials and Methods

### DNA manipulation and protein expression

All polymerase chain reaction (PCR) cloning reactions were performed with High-Fidelity Expand Taq DNA polymerase (Roche Applied Science, Indianapolis, IN, USA). All amplicons were cloned with the Zero-Blunt PCR cloning kit (Life Technologies, Carlsbad, CA, USA), and their identity was verified by sequencing. Full-length cDNA of *hACBD6* and *hLPCAT1* as well as the *C. trachomatis serovar D CT775* gene were cloned into the pAcGFP1 vector (Clontech Laboratories, Mountain View, CA) to yield pFK328, pFK642 and pFK803, respectively. *CT453*, *CT775*, and *hACBD6* were cloned into the pET28a vector (Novagen, Madison, WI, USA) to yield pFK685, pFK686, and pFK136, respectively. Proteins were produced in BL21DE3 cells as previously described (Soupene and Kuypers 2012). Expression was induced by addition of IPTG at a final concentration of 0.5 mmol/L. Cells were harvested after 2–3 h and membrane factions were prepared as previously described (Soupene and Kuypers 2012). The *CT453* gene was also cloned into the GSTag vector (#21877; Addgene, Cambridge, MA, USA) under the control of the *Ptac* promoter to yield pFK814. The two bacterial proteins were insoluble and no activity could be detected after purification under denaturing condition (data not shown).

### 1-acyl-sn-glycerol-3-phosphate acyltransferase activity assays

As described in the result section, activity of CT453 was assayed by monitoring the growth of the *E. coli plsC101*strain (SM2-1, CGSC#7587) at the nonpermissive temperature of 37°C (Coleman 1990). *E. coli plsC101*strain transformed with the GST vector alone or with pFK814 were grown on rich medium (LB broth) supplemented with 100 *μ*g/mL of ampicillin and 10 *μ*mol/L IPTG to induce production of the GST-CT453 recombinant protein. One set of plates was placed at 30°C and a second set at 37°C for 24 h. Assays were also performed on liquid media and growth was monitored at an OD of 600 nm.

### Measurement of lysoPC acyltransferase activity

**Figure 1:**
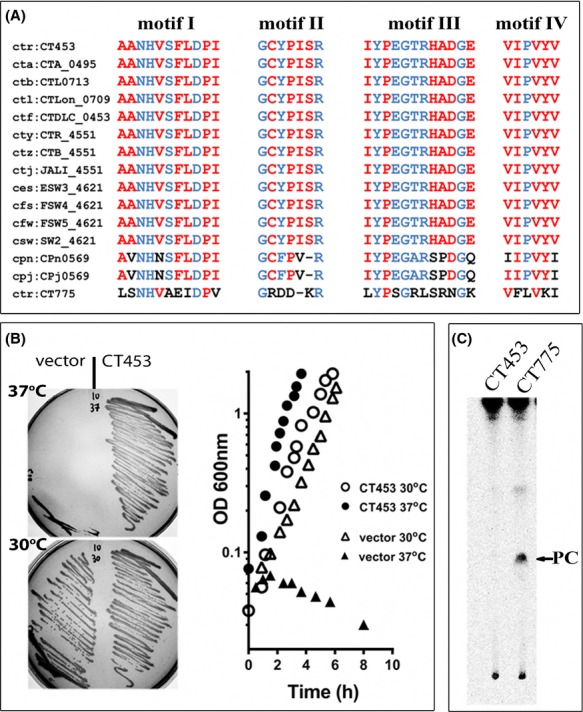
CT453 is a 1-acyl-sn-glycerol-3-phosphate acyltransferase. (A) Amino acids alignment of the predicted four conserved acyltransferase motifs of *Chlamydia trachomatis D* CT453 (top row, GI15605180) and some of the homolog proteins identified in other *Chlamydia* species and serovar, as follows: cta:CTA_0495 (*Chlamydia trachomatis A/HAR-13,* GI76789189); ctb:CTL0713 (*Chlamydia trachomatis L2/434/Bu,* GI166154667); ctl:CTLon_0709 (*Chlamydia trachomatis L2b/UCH-1/proctitis*, GI166155542); ctf:CTDLC_0453 (*Chlamydia trachomatis D-LC,GI385244541*); cty:CTR_4551 (*Chlamydia trachomatis A2497,* GI376282462); ctz:CTB_4551 (*Chlamydia trachomatis B/TZ1A828/OT*, GI237804803); ctj:JALI_4551 (*Chlamydia trachomatis B/Jali20/OT*, GI237802881); ces:ESW3_4621 (*Chlamydia trachomatis E/SW3*, GI389859025); cfs:FSW4_4621 (*Chlamydia trachomatis F/SW4*, GI389858149); cfw:FSW5_4621 (*Chlamydia trachomatis F/SW5*, GI389859901); csw:SW2_4621 (*Chlamydia trachomatis Sweden2*, GI386262810); cpn:CPn0569 (*Chlamydia pneumoniae CWL029*, GI15618480); cpj:CPj0569 (*Chlamydia pneumoniae J138*, GI15836100); ctr:CT775 (*Chlamydia trachomatis D, GI15605508)*. Motifs for *C. trachomatis* LPCAT enzyme, CT775, are shown in the bottom row. Conserved residues defining the four motifs are shown in blue and other conserved residues are in red. (B) Rescue of the growth defect of the *E. coli plsC101* mutant at 37°C by CT453 was monitored on agar plate and in liquid media. Note that the mutant strain transformed with the CT453 construct grew at 30°C and 37°C whereas the control strain only grew at 30°C. (C) Absence of a lysoPC acyltransferase activity of the CT453 enzyme was determined in reactions performed with 5 *μ*mol/L [^14^C]-C_18:1_-CoA in the presence of 20 *μ*mol/L LPC at 37°C for 30 min with 40 *μ*g of clear lysate as described in method. CT775 was included as a control. After separation by thin-layer chromatography, [^14^C]-compounds were detected by phosphoimaging. [^14^C]-PC was used as a migration standard.

Incorporation of [^14^C]C_18:1_-CoA into egg lysoPC by recombinant CT775 protein was determined as previously described (Soupene and Kuypers 2012). Reactions were performed in glass tubes at 37°C in a shaking water bath, in 200 *μ*L of 20 mmol/L potassium phosphate buffer at pH 7.0 with 0.8 mg/mL of Tween-20, containing 20 *μ*mol/L lysoPC and 5 *μ*mol/L [^14^C] C_18:1_-CoA. Reactions were initiated by addition of 4 *μ*g of membrane protein fractions or with 10–40 *μ*g of a cleared lysate, as indicated in the legend of figures. Four time points taken from 0 to 8 min were used to determine the rate of PC formation by CT775. All measurements were performed at least in triplicate. Control experiments were performed with fractions obtained from *E. coli* strains transformed with the empty pET28a vector. Under our growth condition, no detectable *E. coli* acyl-CoA: 1-acyl-lysoPC acyltransferase activity was detected (first lane of Fig. ** **1C) (Soupene and Kuypers 2012). Reactions were stopped and lipids were extracted by the addition of 750 *μ*L of CHCl_3_:MeOH (1/2, v/v), 250 *μ*L of CHCl_3_ and 250 *μ*L of water with vigorous vortexing after each addition. Phases were separated by centrifugation at 1000*g* for 5 min and the lipid-containing chloroform phase was dried down under N_2_. Lipids were dissolved in 20 *μ*L of CHCl_3_:MeOH (2:1, v/v). Samples were applied to TLC silica plates and developed with chloroform/methanol/acetic acid/0.9% NaCl (100:50:16:5, v/v). TLC plates were air-dried for 20 min and exposed to a PhosphoImager screen (Storm 840; Molecular Dynamics, Pittsburgh, PA, USA). Quantification of PC formation was performed with ImageQuant software subtracting the plate background. To determine the effect of divalent cations on CT775 activity the rate of [^14^C]PC formation was measured in absence or presence of 10 mmol calcium chloride in the incubation mixture as previously described (Soupene and Kuypers 2012). To determine the effect of *N*-ethylmaleimide (NEM) on CT775 activity, fractions were first incubated with the chemical for 30 min on ice as previously described (Soupene and Kuypers 2012). Samples were then assayed for acyltransferase activity as described above. To determine the effect of hACBD3 and hACBD6 purified protein (Soupene et al. 2008b) on the activity of CT775, the acyl-CoA substrate [^14^C]C_18:1_-CoA was first incubated with the protein for 20 min at 37°C in the reaction buffer in absence of LPC and CT775. Samples were then assayed for acyltransferase activity as described above. Acylation of LPC by isolated EBs were performed in an axenic culture system developed Omsland et al. (2012). EBs were incubated in presence of 100 *μ*mol/L [^14^C]LPC for 1 h at 37°C in a buffer containing 5 mmol/L KH_2_PO_4_, 10 mmol/L Na_2_HPO_4_, 8 mmol/L KCl, 1 mmol/L MgCl_2_, 100 mmol/L K glutamate, 0.5 mmol/L DTT and 0.5 mmol/L glucose-6 phosphate. Lipids were extracted as above and separated by two-dimensional TLC. Lipids were first resolved in a system of chloroform/methanol/acetic acid/0.9% NaCl (100:50:16:5, v/v). TLC plates were air-dried for 20 min and developed in the second dimension with another acidic system made of chloroform/methanol/acetic acid/water (120:60:16:5.7, v/v). A control silica plate was run with the two standard [^14^C]LPC and [^14^C]PC.

### Cell culture, infection and transfection

HeLa229 cells, obtained from ATCC (CCL-2.1), were maintained in minimal essential medium (MEM alpha; Invitrogen, Carlsbad, CA) containing 10% fetal bovine serum and 2 mmol/L glutamine. For microscopy studies, cells were grown, transfected and infected on 12-mm round coverslips (Electron Microscopy Sciences, Inc., Hatfield, PA) in 24-well plates (Soupene et al. 2012). Cells grown to 70–80% confluence were treated with 500 *μ*L of 45 *μ*g/mL of DEAE-dextran made in PBS for 10 min at room temperature (Scidmore 2005). Cells were washed once with PBS, and infected with 200 *μ*L of *C. trachomatis* strain D (ATCC, VR-885) at a multiplicity of infection of about 1–2 for 1 h at 37°C and 5% CO_2_. Fresh medium was added to the well at a final volume of 1 mL and incubated at 37°C and 5% CO_2_. Infected cells were transfected 4–6 h postinfection with Turbofect (Thermo Scientific, Pittsburgh, PA, USA) according to the manufacturer instruction. Noninfected cells were grown and transfected under the same condition. After 40–44 h, cells were washed twice with PBS and were fixed with 4% paraformaldehyde made in PBS for 30 min. After aspiration, cells were washed twice with PBS and kept at 4°C until use.

### LDs isolation and detection

Production of LDs in treated cells (uninfected or infected, untransfected or transfected) was induced by addition of 100 *μ*mol/L oleic acid complexed with 20 *μ*mol/L defatted BSA to the culture medium 24 h before harvesting. LDs were labeled with 0.5 *μ*g/mL of the fluorescent fatty acids analogue BODIPY558/563 C_12_ (D-3835; Molecular Probes, Carlsbad, CA, USA) complexed with the same oleic acid/BSA mixture as described (Suzuki et al. 2013). Label was chased for 30 min with growth medium and cells were washed and fixed as described above. LDs were isolated and purified as previously described (Liu et al. 2004). Proteins were delipidated and concentrated by acetone precipitation.

### Imaging and staining

Human and bacterial DNA was revealed by staining with 200 *μ*L of 0.5 *μ*g/mL Hoechst 33258 dye (Invitrogen) in PBS for 10 min. Dye was removed by two washes with PBS. Coverslips were mounted on glass slides embedded in FluorSave Reagent (EMD Biosciences, Darmstadt, Germany). Microscopy analysis was performed at room temperature with a Zeiss LSM 710 confocal inverted microscope equipped with a 63× oil objective. Scale bars are indicated in the legend of each figure. Image processing, deconvolution, 3D reconstruction and colocalization analysis were performed with Huygens Essential and Bitplane Imaris Suite package of Scientific Volume Imaging (Hilversum, The Netherlands). Costes thresholds and thresholded Manders’ colocalization coefficients (tMCC) (Dunn et al. 2011) were determined and calculated with Imaris.

### Reverse transcription and real-time PCR

Gene expression analysis was performed using frozen stock of RNA previously collected (Soupene et al. 2012). Expression levels of bacterial *CT453 and CT775* genes were normalized to the values obtained for the *euo* gene (Scidmore-Carlson et al. 1999). The ΔΔCt method was used to determine the expression level values from 2 to 36 h relative to 0 h and the induction profile of *CT775* was plotted relative to the value of *CT453* at *t* = 0 h.

## Results

### *Chlamydia* CT453 is a 1-acyl-sn-glycerol-3-phosphate acyltransferase

Annotation of the *C. trachomatis* genome assigned *CT453* as a putative *plsC* gene. *CT453* was predicted to encode a protein of 217 residues carrying the four conserved acyltransferase motifs (Fig.1A, top row) (Shindou and Shimizu 2009). The protein was insoluble during initial purification steps as a hexahistidine recombinant form (Fig. S1A). A recombinant GST-CT453 protein was also insoluble (not shown) but it was active in vivo and could rescue the growth defect of an *E. coli plsC*^*ts*^ mutant lacking lysophosphatidic acid acyl transferase (LPAAT) activity at 37°C (Figs.1B and S1A). *E. coli* LPAAT enzyme is essential and the *plsC101* mutation allows growth of the mutant cell at low temperature (30°C) but renders an unstable enzyme at the nonpermissive temperature of 37°C and above (Coleman 1990). Both on solid and liquid media, expression of CT453 restored growth of the *plsC101* mutant at 37°C (Fig.1B). These results established that CT453 is a 1-acyl-sn-glycerol-3-phosphate acyltransferase of *C. trachomatis*. Lipids species of *Chlamydia* are characterized by the presence of branched acyl chains at the sn-2 position that are not produced by *E. coli* (Wylie et al. 1997). The activity of CT453 in the *E. coli* membrane suggested that the specific lipid environment is not essential for its activity and that it can use nonbranched acyl-CoA moieties to perform the acylation reaction.

### *Chlamydia* CT775 is a lysophosphatidylcholine acyltransferase

**Figure 2:**
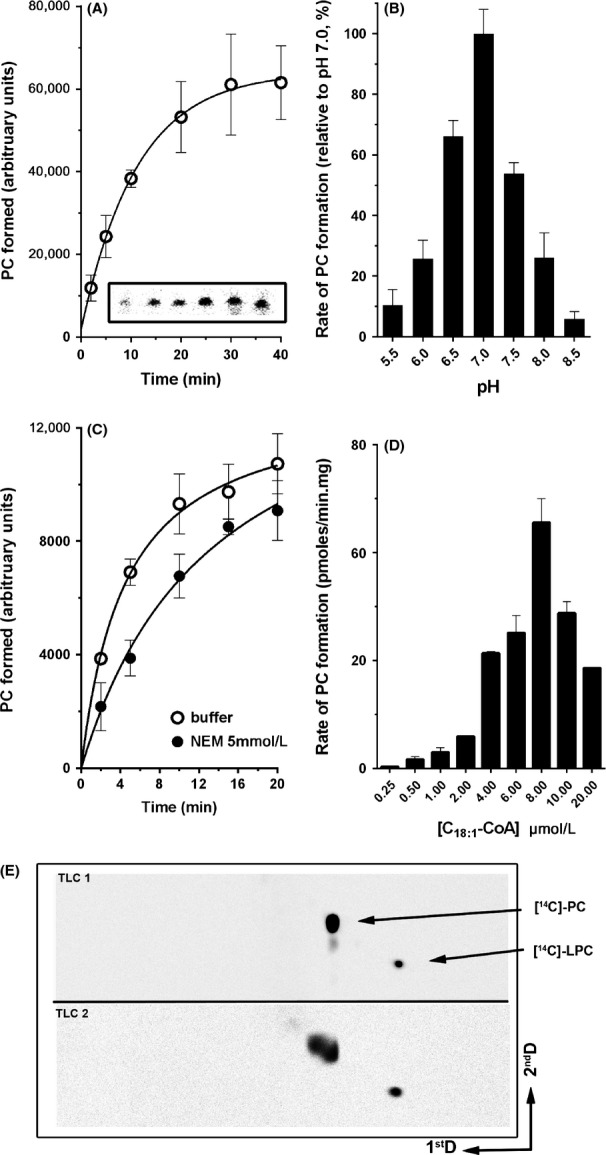
Lysophosphatidylcholine acyltransferase activity of CT775. Measurements of CT775 activity were performed with [^14^C]-C_18:1_-CoA in the presence of 20 *μ*mol/L LPC at 37°C. The standard deviations of at least three different measurements are indicated as error bars. (A) Reactions were performed with 40 *μ*g proteins of a cleared lysate obtained from BL21DE3 strain producing CT775. Inset: image of a representative TLC plate showing production of [^14^C]-PC from 2 to 40 min with 40 *μ*g of cleared lysate as described in the Materials and Methods section. (B) Acylation rates of reactions performed at the indicated pH were calculated between 0 and 8 min in presence of 10 *μ*g of protein. After separation by thin-layer chromatography, the amount of [^14^C]-PC formed during the reactions were quantified by phosphoimaging and the activity rate values were calculated as pmoles of PC formed/*μ*g of protein per min. Values are reported relative to the activity rate value obtained at pH 7.0. (C) Treatment with 5 mmol/L *N*-ethylmaleimide of 10 *μ*g of proteins was performed for 30 min on ice before assaying the acyltransferase activity. The reagent concentration was reduced 20-fold by dilution into the acyltransferase reaction mixture to assay the activity of the treated enzyme. The control sample was incubated for the same period of time and under the same condition in absence of NEM. (D) Acylation rates of 40 *μ*g of proteins incubated in presence of the indicated concentration of [^14^C]-C_18:1_-CoA were calculated from 0 to 8 min. (E) LPCAT activity of EBs was detected in presence of the precursor [^14^C]-LPC at 100 *μ*mol/L as described in the Materials and Methods section. Following incorporation, lipids were extracted and separated on a two-dimensional thin-layer chromatography system, indicated as 1^st^D and 2^nd^D. Position of LPC and PC were determined with [^14^C]-standards run on a separate silica plate (TLC1) under similar condition. Note that two spots co-migrating with standard [^14^C]-PC were obtained with the EBs establishing the incorporation of various species of acyl-CoA donors by the bacterial LPCAT enzyme. NEM, *N*-ethylmaleimide; EB, elementary body; PC, phosphatidylcholine.

PC is the second most abundant lipid in the membrane of *C. trachomatis* and it is scavenged from the host membranes (Wylie et al. 1997). *CT775* encodes a predicted acyltransferase of 253 residues with similarity to CT453 (Fig.1A, last row). *CT775* is also conserved in *Chlamydiae* (not shown: Kyoto Encyclopedia of Genes and Genomes). *Chlamydia* CT775 was successfully produced in *E. coli* associated with the membrane fraction, confirming the prediction that acyltransferases are membrane-bound enzymes (Fig. S1B). In presence of the lysoPC acceptor and C_18:1_-CoA as donor, CT775 produced PC (Figs.1C and 2A). Thus, as was the case for CT453, CT775 can accept nonbranched acyl-CoA species as donor. The LPCAT activity of *C. trachomatis* was also detected when EBs were incubated with [^14^C]-LPC (Fig.2E). When produced in *E. coli,* CT775 reached maximum activity rate at pH 7.0 (Fig.2B). Activity was not affected by treatment with the sulfhydryl-modifier agent *N*-ethylmaleimide (NEM) (Fig.2C), and was not sensitive to the presence of calcium in the reaction (not shown). Under similar conditions, human LPCAT1 activity was decreased 10 and fivefold, respectively (Soupene et al. 2008a; Soupene and Kuypers 2012). The rate of PC formation by CT775 was dependent on the concentration of oleoyl-CoA (Fig.2D). Activity increased to a high value at a concentration of 8.0 *μ*mol/L acyl-CoA but CT775 could not maintain the maximum acylation rate at higher concentrations and activity decreased in a concentration-dependent fashion. Acyl-CoA compounds are powerful detergents and CT775 activity appeared more sensitive to their effect than hLPCAT1 (Soupene et al. 2008a).

Expression data for *CT775* and *CT453* were obtained from analysis of available transcriptome studies that we were able to confirm by RT-qPCR (Fig. S1C). Compared to *CT453*, *CT775* expression was high early in development and stayed high. mRNA level of *CT775* only increased fourfold during *Chlamydia* development (Fig. S1C). These high expression levels early in the infection process were supported by the transcriptome comparison of the elementary and reticulate bodies, which quantified mRNA level of *CT775* 13-fold higher in the EB than in the reticulate body (RB) form of the bacteria (Albrecht et al. 2010). Expression of *CT453* was lower early in development, peaked at 24 h post infection, and increased more than 20-fold during development (Fig. S1C) (Belland et al. 2003). The confirmation that *CT453* and *CT775* represent actively expressed genes in *C. trachomatis* was obtained by detection of matching peptides in the proteome analysis of the bacteria (Saka et al. 2011).

### *Chlamydia* LPCAT enzyme is associated to LDs

**Figure 3:**
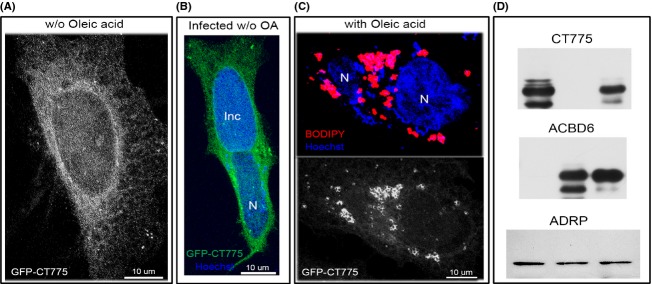
Distribution of *Chlamydia* CT775 is altered by LDs formation. HeLa cells were grown on coverslips and transfected with the GFP-CT775 construct for ∽44 h (A). Cells were infected with *C. trachomatis* strain D 4–6 h before transfection (B and D). Lipid droplets formation was induced by addition of 100 *μ*mol/L of oleic acid 24 h after transfection (C and D). LDs were labeled in vivo by BODIPY C12 (red) for 1 h (C). BODIPY was chased for 30 min and cells were fixed for 30 min in 4% paraformaldehyde made in PBS. Bacteria were detected by staining the *Chlamydia* chromosome with the DNA dye Hoechst 33258 (blue). Images were taken with a Zeiss LSM710 confocal microscope equipped with 63× objective. Nuclei and inclusion are indicated with N and Inc, respectively. Formation of LDs drastically changed distribution of the CT775 tagged protein (A and B vs. C) and quantification analysis determined that 76% of the GFP-CT775 signal co-localized with the BODIPY signal with a tMCC of 0.8481 in (C). (D) Western-blot detection of the GFP-CT775 (58 kDa), GFP-ACBD6 (61 kDa) and of the LDs marker ADRP. Protein extract of HeLa cells transfected with the GFP-CT775 construct (lane 1), of LDs purified from GFP-transfected cells (lane 2), and of LDs purified from GFP-CT775 transfected cells (lane 3) were separated on 10% SDS-PAGE and probed with an anti-GFP antibody (G1544; Sigma). The same antibody was used to detect GFP-ACBD6 in LDs obtained from GFP-transfected cells (lane 1), in protein extract of cells transfected with the GFP-ACBD6 construct (lane 2), and in LDs purified from *Chlamydia*-infected GFP-ACBD6 transfected cells (lane 3). The LDs marker ADRP was detected with an antibody (PA5-25042; ThermoFisher Scientific) in the three LDs samples isolated from GFP-transfected cells (lane 1), from cells transfected with GFP-CT775 (lane 2), and from infected cells transfected with GFP-ACBD6 (lane 3). LDs, lipid droplets; tMCC, thresholded Manders’ colocalization coefficients.

**Figure 4:**
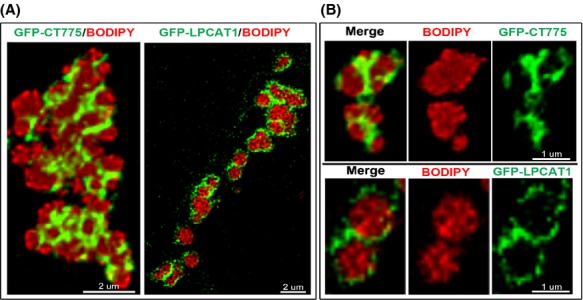
CT775 and hLPCAT1 enzymes are bound to LDs. HeLa cells were grown on coverslips and transfected with GFP-CT775 or with GFP-LPCAT1 (green) for 48 h. LDs production was induced by addition of oleic acid in the growth medium for 24 h and LDs were labeled in vivo for 1 h with BODIPY C12 (red). After cell fixation in 4% paraformaldehyde, DNA was stained with the Hoechst dye (blue). Deconvolved Z-stack merged images of GFP-CT775 and BODIPY and of GFP-LPCAT1 and BODIPY, respectively, are shown in (A). A cropped section of each image showing two LDs is displayed in (B). The merged, and the two single channel views, are presented for CT775 (top) and for hLPCAT1 (bottom). Quantification analysis determined that 75% of GFP-CT775 and 93% of GFP-LPCAT1 co-localized with the BODIPY signal with a tMCC of 0.8969 and of 0.7130, respectively. LDs, lipid droplets; tMCC, thresholded Manders’ colocalization coefficients.

In human cells, a green fluorescent protein (GFP) tagged fusion of CT775 was detected throughout the cytosol surrounding the nucleus (Fig.3A). Infection of the cells by *C. trachomatis serovar D* did not alter the expression pattern of CT775 (Fig.3B). *Chlamydia* was detected by staining the bacterial chromosome with the Hoechst dye, which also stained the host DNA and revealed the nuclei (Fig. S2A) (see Materials and Methods). Upon induction of LD formation, the uniform distribution of GFP-CT775 was lost and most of the GFP signal overlaid with the LDs (Fig.3C), which were detected by in vivo incorporation of the fluorescent lipid analog BODIPY C12 (Suzuki et al. 2013). Further analysis revealed that CT775 was associated with LDs and formed a grape-like tightly packed structure with numerous LDs (Fig.4A). Contrary to hLPCAT1 which is present in the LDs membrane (Moessinger et al. 2011), and was detected as a uniform GFP signal surrounding the droplet, CT775 was detected around the LDs in a noncircular pattern (Fig.4B). As mentioned, the recruitment of CT775 to the membrane of LDs was almost complete, and only a very weak GFP signal was observed not associated with LDs. Under similar condition, the GFP protein was not associated with LDs (Fig. S2B). Presence of CT775 in the LDs was confirmed by immuno-detection of the GFP-tagged protein on LDs purified from transfected cells (Fig.3D). Association of CT775 with the *E. coli* membrane and the acylation of lysoPC that takes place in the *Chlamydia* membrane strongly suggest that in addition to the LDs membrane, CT775 is a membrane-bound LPCAT enzyme of *C. trachomatis*.

### hACBD6 regulates the acylation reaction

**Figure 5:**
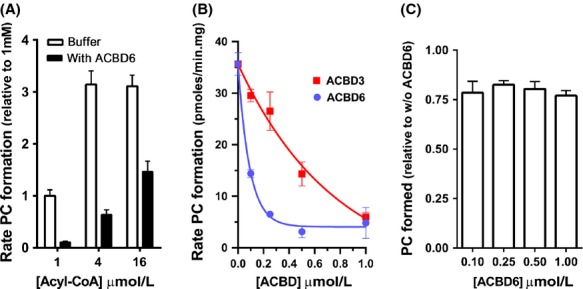
hACBD6 regulates the acylation rate of LPC by CT775. Measurements of CT775 activity were performed with [^14^C]-C_18:1_-CoA in the presence of 20 *μ*mol/L LPC at 37°C. As indicated in each panel, [^14^C]-C_18:1_-CoA was preincubated with purified hACBD3 or hACBD6 before initiation of the acylation reaction by addition of CT775. The standard deviations of at least three different measurements are indicated as error bars. (A) The activity rate values (pmoles of PC formed/*μ*g of protein per min) were calculated from 0 to 6 min with 40 *μ*g proteins of a cleared lysate obtained from the BL21DE3 strain producing CT775. Values are reported relative to the activity rate value obtained at the concentration of 1 *μ*mol/L acyl-CoA w/o hACBD6. Filled bars show rate values obtained in presence of 1 *μ*mol/L purified hACBD6. (B) The activity rate values (pmoles of PC formed/*μ*g of protein per min) were calculated from 0 to 6 min with 4 *μ*g proteins of a membrane preparation obtained from the BL21DE3 strain producing CT775. Measurements were performed with 1 *μ*mol/L [^14^C]-C_18:1_-CoA preincubated with the indicated concentration of purified hACBD3 and hACBD6. (C) Amounts of PC formed after 2 h incubation of CT775 with 1 *μ*mol/L acyl-CoA and with the indicated concentrations of hACBD6 are reported relative to the value obtained in reaction performed in absence of hACBD6. Note that the total amount of PC formed after 2 h of incubation was not dependent of the initial acylation rate. PC, phosphatidylcholine.

Members of the acyl-CoA binding family (ACBD) are thought to maintain high concentrations of acyl-CoA outside membranes and protect the various acylCoA-utilizing enzymes from the detergent nature of their substrate (Burton et al. 2005; Fan et al. 2010). In human *Chlamydia-*infected cells, hACBD6 was recruited into the lumen of the inclusion suggesting a role of the acyl-CoA carrier in some aspect of lipid metabolism of *Chlamydia* (Soupene et al. 2012). The rates of PC formation by CT775 were measured in vitro by following transfer of [^14^C] C_18:1_-CoA onto lysoPC in presence of purified hACBD6 protein. At a molar acyl-CoA/hACBD6 ratio of 1, the incorporation rate was decreased to less than 20% of the rate obtained in absence of hACBD6 (Fig.5A). As expected for a reaction limited by substrate availability rather than inhibition by the protein, the acylation rates were increased when concentration of acyl-CoA was increased. It should be noted that due to the concentration-dependent formation of micellar structures by the substrate, which would be affected by the two proteins and by the presence of the lysoPC donor, substrate titration of such complex mixtures was not attempted. Instead, concentration of the protein was varied at a fixed substrate concentration. At a limiting substrate concentration of 1 *μ*mol/L, 0.1 *μ*mol/L of hACBD6 was enough to reduce the acylation rate by 50% (Fig.5B). Under these conditions, 0.1 *μ*mol/L hACBD3 decreased the rate of CT775 by only 15%. At a 1 to 1 substrate/protein ratio, hACBD3 also reduced acylation rates down to 20% as did hACBD6 (Fig.5B).

As mentioned, acylation by CT775 was dependent on the concentration of acyl-CoA and decreased at concentrations higher than 8 *μ*mol/L (Fig.2D). The acylation rates calculated in the first 8 min of the reaction were lower in hACBD6 presence, but CT775 activity steadily increased with the acyl-CoA concentrations even under conditions that were inhibitory in absence of hACBD6 (Fig.5A). The *apparent* contradictory findings of an inhibition of the acylation rate by hACBD6 and the lack of inhibition by high substrate concentration suggested that the acyl-CoA binding protein modulated the acylation reaction by controlling the substrate availability to the enzyme. To provide further evidence of its modulatory function, the amount of PC produced by CT775 in presence of hACBD6 was measured in incubations of 2 h, substantially longer than those used to determine the acylation rate (8 min). Under such conditions, the total amount of ^14^C-PC that could be extracted from the microsomal membranes in the presence of hACBD6 was about 80% of the amount extracted in its absence. However, production of PC did not decrease as the concentration of hACBD6 increased (Fig.5C). Even under condition of very slow acylation rate (1 to 1 molar ratio), the amount of PC produced was not lower than the amount detected at faster acylation rate (Fig.5C). Those are strong lines of evidence supporting the conclusion that hACBD6 acted as an acyl-CoA buffering regulator allowing the controlled release of the substrate to the bacterial acyltransferase. The kinetic parameters of the reaction performed by the hACBD6/acyl-CoA/ctLPCAT/LPC system cannot be determined in absence of a quantitative analysis of *Chlamydia* lipids inside the inclusion.

### hACBD6 distribution is not altered by LDs formation

**Figure 6:**
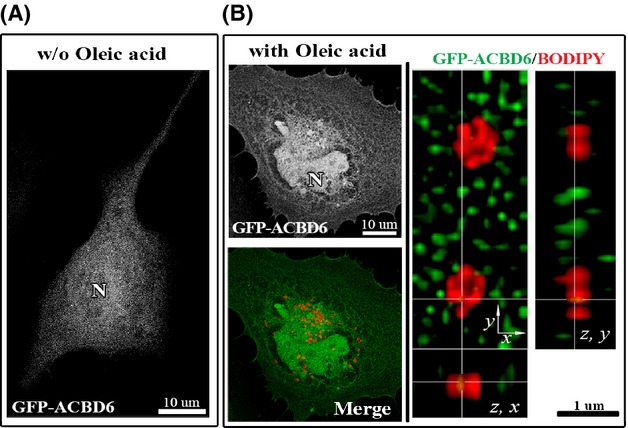
hACBD6 is not associated to LDs in HeLa cell. HeLa cells were grown on coverslips and transfected with GFP-ACBD6 (green) for 48 h (A). LDs production (B) was induced by addition of oleic acid in the growth medium for 24 h and LDs were labeled with BODIPY C12 (red). After cell fixation in 4% paraformaldehyde, DNA was stained with the Hoechst dye (blue).) Cropped orthogonal (*z*, *x*) and (*z*, *y*) views of the deconvolved *Z*-stack merged image is shown on the right. Note the lack of overlay of the GFP and BODIPY signal (tMCC of 0.0431). LDs, lipid droplets.

In human cells, a GFP-ACBD6 was detected throughout the cytosol and the nucleus (Fig.6A). Another member of the family, hACBD1 (DBI), is also known to transit into the nucleus (Elholm et al. 2000). The nuclear localization of the protein was confirmed by immuno-detection on fixed cells and by western-blotting of protein nuclear extract (Soupene et al. 2012). Contrary to CT775 which was almost completely recruited to the LDs (Fig.3C), the distribution of hACBD6 was similar in cells producing LDs as compared to nonstimulated cells (Fig.6B). hACBD6 was still present in the cytosol and nucleus of the cells and it was not associated to LDs.

### *Chlamydia* development triggers association of hACBD6 to LDs

**Figure 7:**
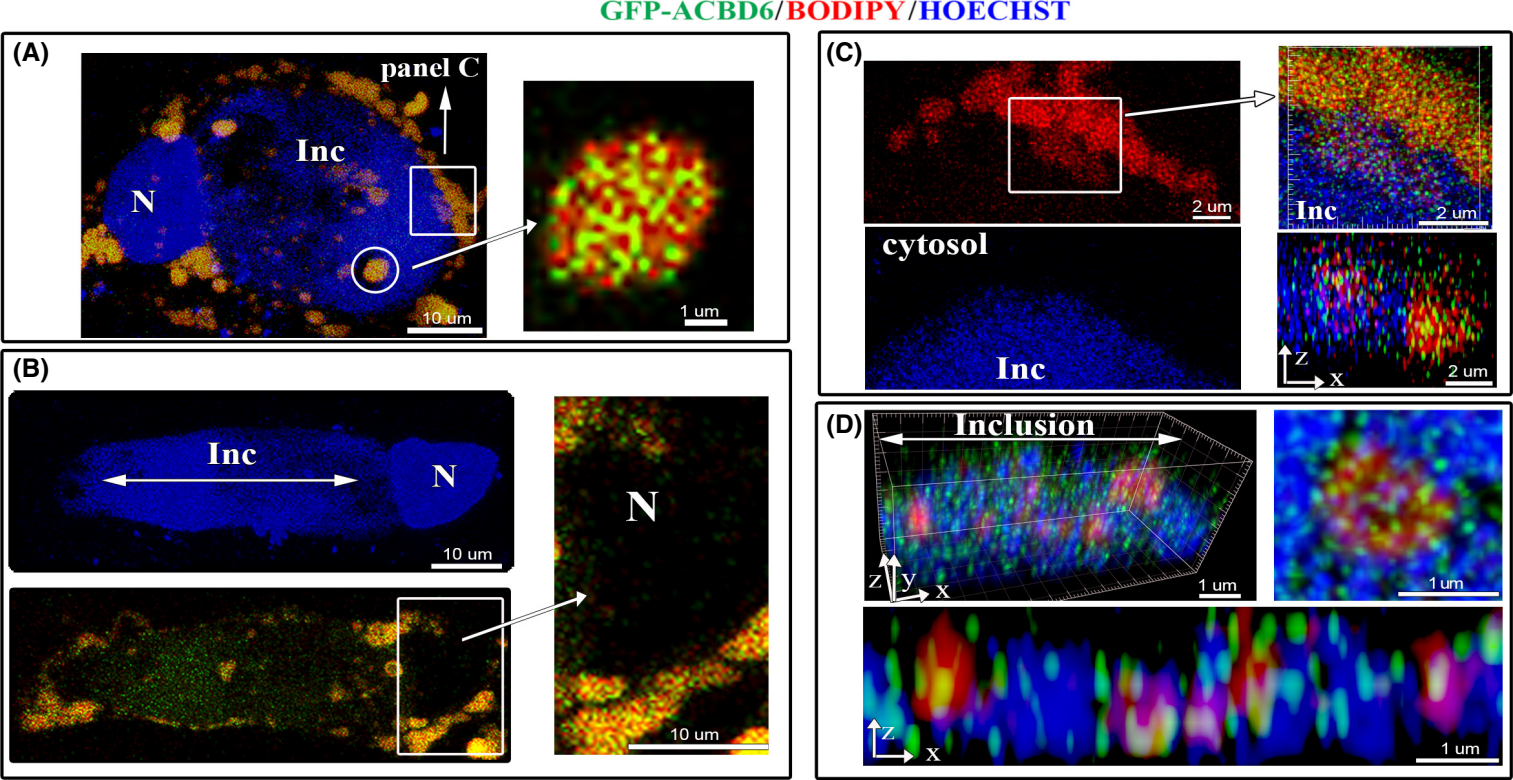
Relocation of hACBD6 to LDs and into the inclusion during *Chlamydia* development. HeLa cells were grown on coverslips, infected with *C. trachomatis* 6 h prior transfection with GFP-ACBD6. LDs production was induced by addition of oleic acid in the growth medium for 24 h and LDs were labeled with BODIPY C12 (red). After cell fixation in 4% paraformaldehyde, DNA was stained with Hoechst dye (blue). Nuclei and inclusion are indicated with N and Inc, respectively. (A) A merged image of an infected cell accumulating LDs with a single large inclusion. Note that in the cytosol, the GFP signal overlaid with the BODIPY signal. Inset: cropped view of a deconvolved *Z*-stack merge image of a lipid droplet associated with GFP-ACBD6 (92% co-localization with a tMCC of 0.9411). (B) An infected cell with a large inclusion is shown in the single blue channel (Hoechst staining, top) and is shown as a merged image of the signal of GFP-ACBD6 and BODIPY (green and red, bottom). Note the detection of the GFP signal in the lumen of the inclusion. Inset: cropped view showing the absence of the GFP signal in the nucleus of the infected cell. (C) A cropped view of a section of the cell shown on (A) which captured the apparent migration of the LDs (red signal) inside the large inclusion (blue area). Cropped orthogonal views confirmed that the GFP and BODIPY signal were detected overlaid on the cytosolic side surrounding the inclusion as well as in the lumen of the inclusion. (D) Cropped orthogonal *x*, *y*, *z* view of the inside of an inclusion is shown as a merge image of the *Chlamydia* chromosome (blue), lipid droplets (red) and ACBD6 (green). A *x*, *z* view is shown at the bottom and a cropped view of a single LDs with bound GFP-ACBD6 inside the inclusion is shown on the right (91% co-localization with a tMCC of 0.9282). LDs, lipid droplets.

Infected cells were stimulated for production of LDs and distribution of GFP-ACBD6 was determined. Compared to un-infected cells (Fig.6B), GFP-ACBD6 was almost exclusively localized with LDs in the cytosol of *Chlamydia-*infected cells (Fig.7A–C). GFP-ACBD6 was also detected inside the inclusion and could no longer be observed in the nucleus of infected cells (Fig.7B). GFP protein was not observed in the inclusion of infected cells (Fig. S2C). Presence of hACBD6 on LDs purified from infected cells was confirmed by immuno-detection (Fig.3D). The movement of hACBD6 out of the nucleus into the inclusion is an event which occurs progressively during development of the *Chlamydia* vacuole and is achieved ∽24 h post infection (Soupene et al. 2012). The association of the protein to the LDs was also only observed late in development (not shown). In infected cells, numerous LDs could be detected inside the inclusion (Fig. S2D). Cropping of high resolution deconvolved serial *Z*-stack images also revealed caption showing LDs apparently crossing into the inclusion in association with GFP-ACBD6 (Fig.7C inset). Inside the inclusion, hACBD6 was detected throughout the lumen as well as associated with numerous LDs (Fig.7D). Thus, infection of the cells by the pathogen triggered association of hACBD6 to the LDs, which were mobilized inside the inclusion, presumably to the benefit of the bacterial acyltransferase enzymes.

## Discussion

LDs are organelles involved in diverse cellular processes. Their function in lipid metabolism is the transport and storage of the neutral lipids, triacylglyceride, and cholesterol ester in the cell (Cermelli et al. 2006; Sturley and Hussain 2012; Pol et al. 2014). The monolayer assembly of phospholipids in the surface provides a hydrophilic interface with the surrounding medium. This unique structural characteristic allows entities such as LDs and lipoprotein particles to maintain a stable hydrophobic lumen with a hydrophilic surface exposed to the cytosol or plasma. Although, lipoprotein particles contain few proteins, hundreds of proteins of various functions are associated with the LDs (Cermelli et al. 2006).

The involvement of LDs in the development of pathogenic bacteria (e.g., *Mycobacterium, Chlamydia*) (Cocchiaro et al. 2008; Elamin et al. 2012), viruses (HCV, DENG, rotavirus) (Cheung et al. 2010; Heaton and Randall 2011; Herker and Ott 2011) and parasites (*Plasmodium, Toxoplasma*) (Jackson et al. 2004; Nishikawa et al. 2005) are well documented. Following the entry of the lipoviroparticles of the hepatitis C virus, LDs are hijacked to confer a site of assembly of new particles and the encapsidation of the virion RNA (Herker and Ott 2011). The capsid protein of the dengue virus interacts with LDs and the energy required to support its replication is generated by lipophagy of the LDs (Heaton and Randall 2011). LDs accumulate in the cytosol of *Toxoplasma gondii* and in the food vacuole of *Plasmodium falciparum* (Jackson et al. 2004; Nishikawa et al. 2005). In the parasite vacuole of infected erythrocytes, LDs appear to prevent the accumulation of toxic levels of heme, generated by degradation of hemoglobin (Herker and Ott 2012). In cells infected by *C. trachomatis*, LDs produced by the host cells interact with the inclusion membrane and following their association with bacterial proteins are translocated inside the parasitophorous vacuole (Kumar et al. 2006; Cocchiaro et al. 2008). We are reporting that a human protein, hACBD6, becomes associated to LDs prior their translocation into the inclusion.

LDs are also implicated in defense mechanisms of the infected cells against pathogens and their destruction might be an attempt of the pathogen to evade the immune system of the host. The protective property of LDs is attributed to their association with proteins and molecules that are toxic to intra-cellular pathogens. Several histone proteins are naturally associated with LDs and histones-bound LDs are potent antibacterial vesicles. Bacterial lipopolysaccharides disrupt the interaction of histones with their receptor in the LDs membrane and the unbound histone kills the pathogen (Anand et al. 2012). Intriguingly, a compound developed for its anti-viral property against the dengue virus was shown to bind with cytosolic LDs in *C. trachomatis*-infected cells and to strongly inhibit the growth of the bacterial pathogen (Sandoz et al. 2014).

**Figure 8:**
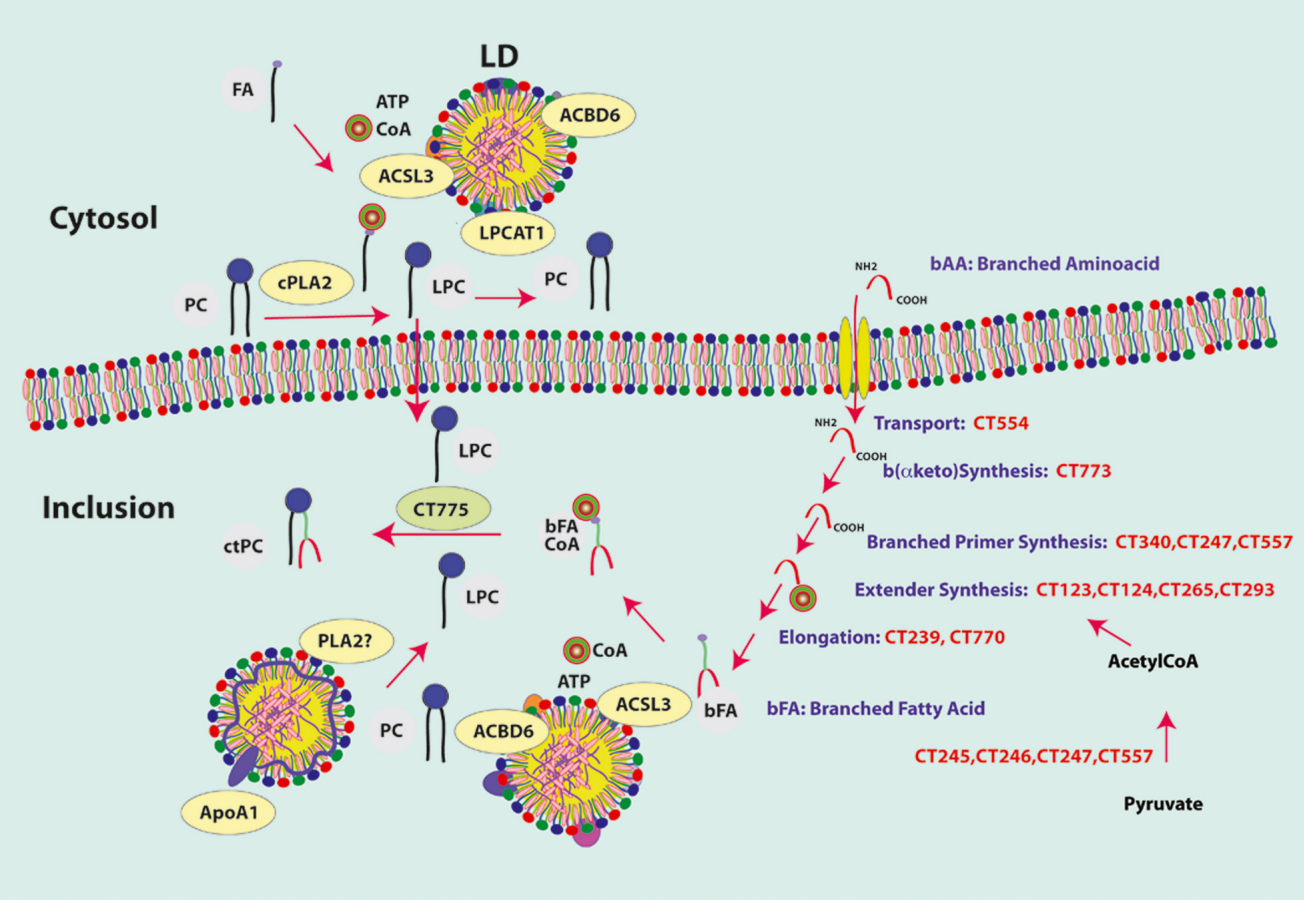
Proposed model of host PC remodeling in *Chlamydia*-infected cells. The branched amino acids (bAA) isoleucine, valine, and leucine are imported into the inclusion by the bacterial transporter BrnQ (CT554). Branched *α* keto acids (*α*-keto-methylvalerate, *α*-keto-isovalearte, *α*-keto-isocaproate) are produced by deamination of bAAs by leucine dehydrogenase (Ldh; CT773). The branched primers (2-methylbutyryl-CoA, isobutyryl-CoA, isovaleryl-CoA) are produced by the Branched *α* Keto Dehydrogenase complex (PdhA_B, PdhC, LpdA; CT340, CT247, CT557) from the b(*α*-keto) intermediates. The extender malonyl-CoA is produced by the acetyl-CoA carboxylase complex (AccA, AccB, AccC, AccD; CT123-124-265-293) from acetyl-CoA. Acetyl-CoA is produced from pyruvate by the pyruvate dehydrogenase complex (PdhA, PdhB, PdhC, LpdA; CT245, CT246, CT247, CT557) and is also the primer for de novo synthesis of straight-chain fatty acids. Branched-fatty acids (bFA) present at the *sn-2* position of *Chlamydia* lipids are generated by elongation by FabF (CT770) and FabH (CT239) of the branched primers. Branched FAs are then activated by the human long-chain acyl-CoA synthetase ACSL3 and transferred on lysoPC by the bacterial LPCAT enzyme (CT775). Human acyl-CoA binding protein ACBD6 buffered the lumen concentration of acyl-CoAs to prevent inhibition of CT775. The lysoPC precursor can be obtained by attack of PC of the inclusion membrane and of LDs by cytosolic PLA2 enzyme. LPC could be generated inside the inclusion from PC of the LDs transported in the inclusion by the lipase activity of an unidentified PLA2 enzyme or of the HDL particles recruited in the inclusion. Acyl chains are pictured in green, choline head group in blue, and CoASH in brown. hACBD6, hACSL3, and hLPCAT1 proteins are shown bound to the lipid monolayer surrounding the LDs. A HDL particle is indicated associated to ApoAI lipoprotein. PC, phosphatidylcholine; LDs, lipid droplets.

In *C. trachomatis*-infected cells, at least three different types of vesicles, multivesicle bodies, LDs and HDL particles, produced in the cytosol have been shown to cross the membrane of the inclusion (Beatty 2006; Cocchiaro et al. 2008; Cox et al. 2012). We described that this is an important step to achieve the remodeling of host lipids into specific bacterial moieties. LD-bound human proteins are translocated into the inclusion to support the limited capability of *Chlamydia*. The lack of a cell-free culture system and the fragility of the inclusion outside a cell (Matsumoto 1981), have made it very difficult to achieve detailed biochemical characterization of lipid metabolic processes that take place inside the inclusion. However, the results obtained from labeling metabolic experiments combined with the requirements of the bacteria for survival and proliferation, allow the proposal of a working model supported by lipidomic, proteomic, and transcriptomic analysis as depicted in Figure8 (Hackstadt et al. 1995, 1996; Wylie et al. 1997; Hatch and McClarty 1998; Robertson et al. 2009; Albrecht et al. 2010; Saka et al. 2011). It was proposed that cPLA2 on the cytosolic side of the inclusion membrane (Su et al. 2004; Du et al. 2011), generates the lysoPC acceptor from PC molecules brought to the inclusion membrane by LDs and by proximity contact with the host ER membrane. The ER is the site of de novo synthesis of PC. In coordination with the formation of acylCoA from fatty acid (FA), hLPCAT1 can reacylate lysoPC to PC and, supports the expansion and the lipid remodeling of the inclusion membrane. LysoPC can also flip from the cytosolic side to the lumen side of the inclusion membrane where it is acylated by the bacterial LPCAT enzyme (CT775) with bacterial-specific branched-chain fatty acids (bFA) to generate unique bacterial PC molecular species. The formation of the branched-chain acyl-CoA (bFA-CoA) is poorly documented but it can be deduced by analogy with reports of other bacteria that are known to synthesize bFA. The large majority of bFA molecules only differ from “straight-chain” FA by a methyl group at n-2 or n-3 position of the acyl chain with different lengths (Kaneda 1991; Wylie et al. 1997; Zhu et al. 2005; Sun et al. 2012). bFAs cannot be obtained directly by methylation of FA. The de novo synthesis of bFA requires the extension of the acyl chain by condensation of malonyl-CoA on primer molecules other than the acetyl-CoA primer. These primers are generated from branched amino acid (bAA) instead of pyruvate. Bacteria that produce both types of acyl chains have a distinct, albeit similar, multi-subunit large enzymatic complex for the synthesis of the branched primers. Pyruvate dehydrogenase (PDH) produces acetyl-CoA from the *α* keto acid pyruvate (Stephens et al. 1983) and the branched-chain *α* keto acid dehydrogenase complex (BKD) produced isobutyryl-CoA, isovaleryl-CoA, and methylbutyryl-CoA from branched *α* keto acids obtained by deamination of the branched amino acids valine, leucine and isoleucine, respectively (Kaneda 1991; Zhu et al. 2005). The PDH complex of *C. trachomatis* is PdhA (CT245), PdhB (CT246), and PdhC (CT247). bAAs are imported into the inclusion by the bacterial ABC transporter BrnQ (CT554) (Braun et al. 2008). Deamination of valine, leucine and isoleucine by CT773 (Ldh) generates the branched *α* keto acid intermediates. No *bkd* genes are annotated in *C. trachomatis* genome but a single gene, *CT340* (*pdhA_B*), encodes both the *α* and *β* subunits of the E1 decarboxylase unit of the BKD complex, which are encoded by the two separated genes *pdhA* and *pdhB* in the PDH enzyme (Fig. S3). A dedicated dihydrolipoamide acetyltransferase E2 unit (PdhC) for the BKD complex was not identified and it is probably shared with the PDH complex. *Listeria monocytogenes* and *Bacillus subtilis* have a dedicated complex for synthesis of branched primers but PDH can accept branched *α* keto acids as substrate (Kaneda 1991; Zhu et al. 2005). Given its compact genome and opportunistic capability, *Chlamydia* might have evolved de novo synthesis of bFA by duplication/fusion of genes of the essential PDH pathway. In both pathways, the lipoamide cofactor is regenerated by the same subunit LpdA (CT557) and the common extender molecule, malonyl-CoA, is produced by AccA, AccB, AccC, and AccD (CT123, CT124, CT265, CT293). The condensation/elongation reaction is also common to both pathways and is performed by FabH (CT239) and FabF (CT770). No fatty acid “activating” enzyme, long-chain acyl-CoA synthetase (FadD), is annotated in *C. trachomatis* genome. A *fadD* gene is predicted in the genome of the related *Waddlia chondrophila*. We have shown that the human LD-bound ACSL3 is recruited into the inclusion where it uses ATP and Coenzme A in the final step to produce bacterial bFA-CoA.

The host acyl-CoA carrier protein hACBD6 binds LDs during development of *Chlamydia* and is translocated into the inclusion. In vitro, hACBD6 prevented inhibition of the *Chlamydia* acyltransferase CT775 by acyl-CoAs and sustained the formation of PC. Stimulation of the production of LDs did not affect the distribution of GFP-ACBD6, but infection of the cells by *C. trachomatis* resulted in drastic alteration of the location of hACBD6. This finding is the first evidence that *Chlamydia* induces an unknown mechanism that triggers the association of hACBD6 to the LDs. The bacterial LPCAT enzyme was also associated to LDs. Interestingly, the human LPCAT enzyme hLPCAT1 which is associated with LDs in the cytosol was not detected in the inclusion (Soupene et al. 2012). The removal of hLPCAT1 from the membrane of LDs crossing into the inclusion appears specific since other known LD-bound enzyme such as hACSL3 (Fujimoto et al. 2004) was transported into the vacuole (Soupene et al. 2012). Branched and straight-chain acyl-CoA molecules are present inside the inclusion. The activity of a straight-chain acyl-CoA:acyltransferase such as hLPCAT1 could result in the depletion of the lysoPC acceptor inside the inclusion and compete with the formation of the branched-chain PC molecules by the bacterial LPCAT enzyme. In this context, the removal of hLPCAT1 and the loading of hACBD6 on the LDs crossing the inclusion membrane would prevent substrate limitation (LPC) and substrate inhibition (acyl-CoAs) of the bacterial LPCAT.

In addition to lysoPC, the host PC molecules are also imported into the inclusion as part of the monolayer membrane of the LDs and HDL particles translocated into the vacuole. The inclusion membrane also contains PC molecules that could be used to support lipid metabolism of the bacteria. Removal of that lipid should not alter the integrity of the inclusion since the membrane would be replenished by PC molecules from the LDs and ER membrane. Inside the inclusion, a lipase is required to de-acylate the host PC, and to generate the precursor for production of *C. trachomatis*-specific PC. An activated form of a cytosolic PLA2 enzyme is associated with the inclusion membrane but it does not appear to act on the lumen side of the membrane. *C. trachomatis* has five genes encoding PLD enzymes (CT154, CT155, CT156, CT157, CT284) (Nelson et al. 2006) but no enzyme with a PLA_2_ activity is predicted in the genome. An enzyme from the host cell with phospholipase activity could be present in the inclusion, but has not been identified. In absence of cholesterol, the lipase activity associated with HDL particles, present in the inclusion, would transfer the sn-2 acyl of PC on a molecule of H_2_O and generate a fatty acid and lysoPC (Subbaiah et al. 1985). Thus, lysoPC could be generated inside the inclusion, and sustain production of this essential bacterial lipid.

The complete transfer of hACBD6 from the nucleus of the infected cell into the *Chlamydia* vacuole, and its association to LDs, may represent a mechanism that the pathogen employs to control the fate of the host cell. The role and regulatory function of acyl-CoA binding proteins on processes as diverse as maintaining pluripotency of neuronal stem cells and assuring replication of viruses in mammals (Fan et al. 2010), and other various function in plants (Xiao and Chye 2011) are well documented. The interaction of these acyl-CoA binding proteins to various membranes and proteins are probably regulated by their ligands, acyl-CoA. As shown by the growth inhibitory effect of a drug preventing binding of acyl-CoA to an ACBD protein of the human pathogen *Cryptosporidium parvum* (Fritzler and Zhu 2012), disruption of the binding property of hACBD6 could prevent its association to LDs, which would affect the generation of essential lipids and suppress growth of the *C. trachomatis*.

## Conflict of Interest

None declared.

## Supporting Information

**Figure S**1**.** Expression, production and purification of *Chlamydia* acyltransferases and human ACBD proteins. (A) Protein samples were obtained from the BL21DE3 strain carrying the vector and the hexahistidine-CT453 construct before and after addition of IPTG at a final concentration of 0.5 mmol/L for 3 h. Approximately 8 *μ*g of proteins were loaded in each lane and were separated on a SDS-PAGE 12% gel. The molecular mass standard (Precision Plus Protein standards, Bio-Rad) is shown on the right. The gel was stained with GelCodeBlue (ThermoFisher). A band of the predicted molecular mass of 24 kDa was detected for CT453 in the induced lane. Bottom inset: immuno-detection of the hexahistidine protein with an anti-histidine antibody (15165; ThermoFisher Scientific) in a crude extract and in the insoluble fraction. The prestained molecular mass standard (Precision Plus Protein Dual-Stained; Bio-Rad) is shown on the right. Top inset: proteins obtained from the *plsC101* mutant carrying the GST-CT453 construct (lane 2) and the GST vector (lane 3) grown at 30°C were separated and stained with GelCodeBlue. The molecular mass standard (Precision Plus Protein standards; Bio-Rad) is shown in lane 1. (B) Soluble and membrane fractions were obtained from the BL21DE3 strain producing CT775. Measurements of the formation of PC with 4 *μ*g proteins obtained from the soluble and membrane fraction were performed with 5 *μ*mol/L [^14^C]-C_18:1_-CoA and 20 *μ*mol/L LPC at 37°C. Inset: proteins were separated as in (A) electrotransfered on a PVDF membrane, and the hexahistidine tag of CT775 was detected with an anti-His antibody. The prestained molecular mass standard (Precision Plus Protein Dual-Stained; Bio-Rad) is shown on the left. (C) RT-qPCR analysis was performed as described in the section. At the indicated time, total RNAs were isolated and cDNAs were synthesized with random primers. mRNA expression levels of the *C. trachomatis* genes *CT453*, *CT775* were normalized to the expression level of the *euo* gene. Expression level obtained for *CT453* at time point 0 h was arbitrary set at a value of 1 and all other values were calculated relative to it. Result of a single experiment is shown. (D) Coomassie-stained SDS-PAGE gel of various fractions obtained during the affinity purification of human hexaHistidine-ACBD3 (top) and -ACBD6 (bottom) proteins as previously described (Soupene et al. 2008b).

**Figure S**2**.** Detection of *C. trachomatis,* of lipid droplets and of GFP protein. (A) This panel was generated using data previously collected and published (Soupene et al. 2012). HeLa cells infected with *Chlamydia trachomatis* D were fixed after 24 h infection and stained with a rabbit polyclonal antibody against the bacterial protein IncA which is located in the inclusion membrane. The image shows two infected cells. One cell has a single large inclusion and the second cell has two inclusions of medium size (indicated as Inc) and two smaller inclusions (indicated by two arrows). As presented in the result section, staining of the chromosome of *Chlamydia* by the Hoechst DNA dye allows detection of the inclusions as well as the nuclei. Moreover, Hoechst staining was more efficient than IncA-staining in detecting very small inclusion (right image). This methodology (detection the nuclei and *Chlamydia* in the blue channel, the GFP-tagged protein in the green channel and the lipid droplets in the red channel) was adopted to maximize signal intensity while avoiding signal overlap. (B) Images obtained with HeLa cells transfected with the AcGFP control vector (green) and grown in presence of oleic acid to induce production of lipid droplet (red). Inset shows the cropped view of a single LDs. GFP signal was not detected associated with LDs. (C) Images obtained with HeLa cells infected with *C. trachomatis* (blue) and transfected with the AcGFP control vector (green). The GFP protein encoded by this vector is detected in the nucleus of cell but it is not detected inside the inclusion. Note that this GFP protein is not translocated in the nucleus when fused to nonnuclear protein such as hLPCAT1. (D) Images obtained with HeLa cells infected with *C. trachomatis* (blue) and grown in presence of oleic acid to induce production of lipid droplet (red). 3D rendering of the inside of an inclusion confirming the presence of LDs is shown on the left and orthogonal *x*, *y*, *z* views on the right.

**Figure S**3**.** Residues alignment of the subunits of the predicted E1 component of the PDH and BKD complexes of *C. trachomatis*. Alignment of the *α* (PdhA; CT245) and *β* (PdhB; CT246) subunits of the predicted E1 component of the Pyruvate Dehydrogenase (PDH) complex and of the fused *α*/*β* single subunit (PdhA_B; CT340) of the Branched *α*Keto Decarboxylase (BKD) complex was generated by the MultAlin program. A cartoon representation of the three subunits is shown in the inset.
