# Supplementary figures and images for: Remodeling of host phosphatidylcholine by Chlamydia acyltransferase is regulated by acyl-CoA binding protein ACBD6 associated with lipid droplets
Source: Microbiologyopen. 2015 Jan 21;4(2):235–51. doi: 10.1002/mbo3.234 (PMC4398506; doi:10.1002/mbo3.234)

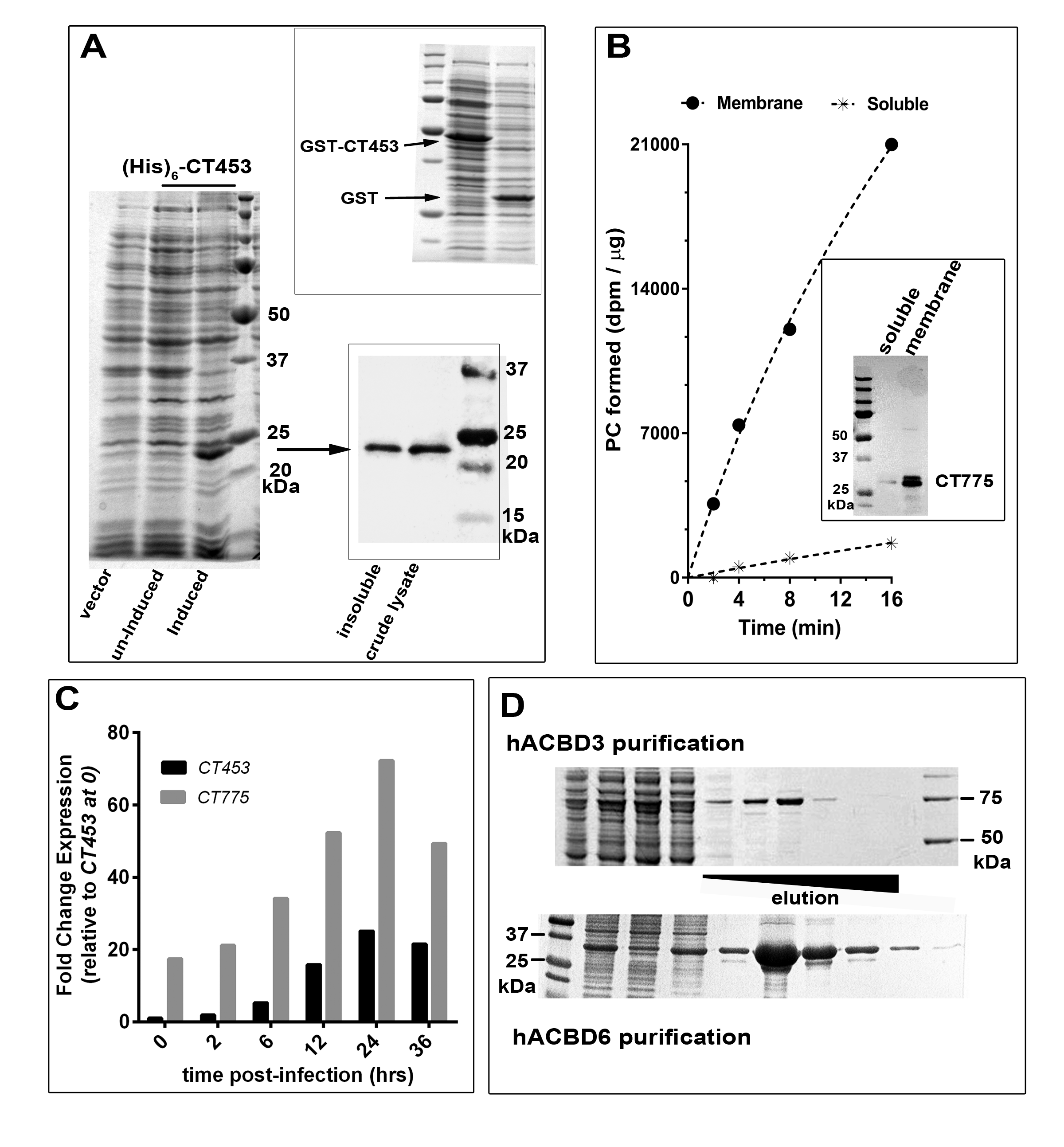

Supplement: Supplementary file 1 — Figure S1. Expression, production and purification of Chlamydia acyltransferases and human ACBD proteins. (A) Protein samples were obtained from the BL21DE3 strain carrying the vector and the hexahistidine-CT453 construct before and after addition of IPTG at a final concentration of 0.5 mmol/L for 3 h. Approximately 8 μg of proteins were loaded in each lane and were separated on a SDS-PAGE 12% gel. The molecular mass standard (Precision Plus Protein standards, Bio-Rad) is shown on the right. The gel was stained with GelCodeBlue (ThermoFisher). A band of the predicted molecular mass of 24 kDa was detected for CT453 in the induced lane. Bottom inset: immuno-detection of the hexahistidine protein with an anti-histidine antibody (15165; ThermoFisher Scientific) in a crude extract and in the insoluble fraction. The prestained molecular mass standard (Precision Plus Protein Dual-Stained; Bio-Rad) is shown on the right. Top inset: proteins obtained from the plsC101 mutant carrying the GST-CT453 construct (lane 2) and the GST vector (lane 3) grown at 30°C were separated and stained with GelCodeBlue. The molecular mass standard (Precision Plus Protein standards; Bio-Rad) is shown in lane 1. (B) Soluble and membrane fractions were obtained from the BL21DE3 strain producing CT775. Measurements of the formation of PC with 4 μg proteins obtained from the soluble and membrane fraction were performed with 5 μmol/L [14C]-C18:1-CoA and 20 μmol/L LPC at 37°C. Inset: proteins were separated as in (A) electrotransfered on a PVDF membrane, and the hexahistidine tag of CT775 was detected with an anti-His antibody. The prestained molecular mass standard (Precision Plus Protein Dual-Stained; Bio-Rad) is shown on the left. (C) RT-qPCR analysis was performed as described in the section. At the indicated time, total RNAs were isolated and cDNAs were synthesized with random primers. mRNA expression levels of the C. trachomatis genes CT453, CT775 were normalized to the expression level of the e [file mbo30004-0235-sd1.tif]

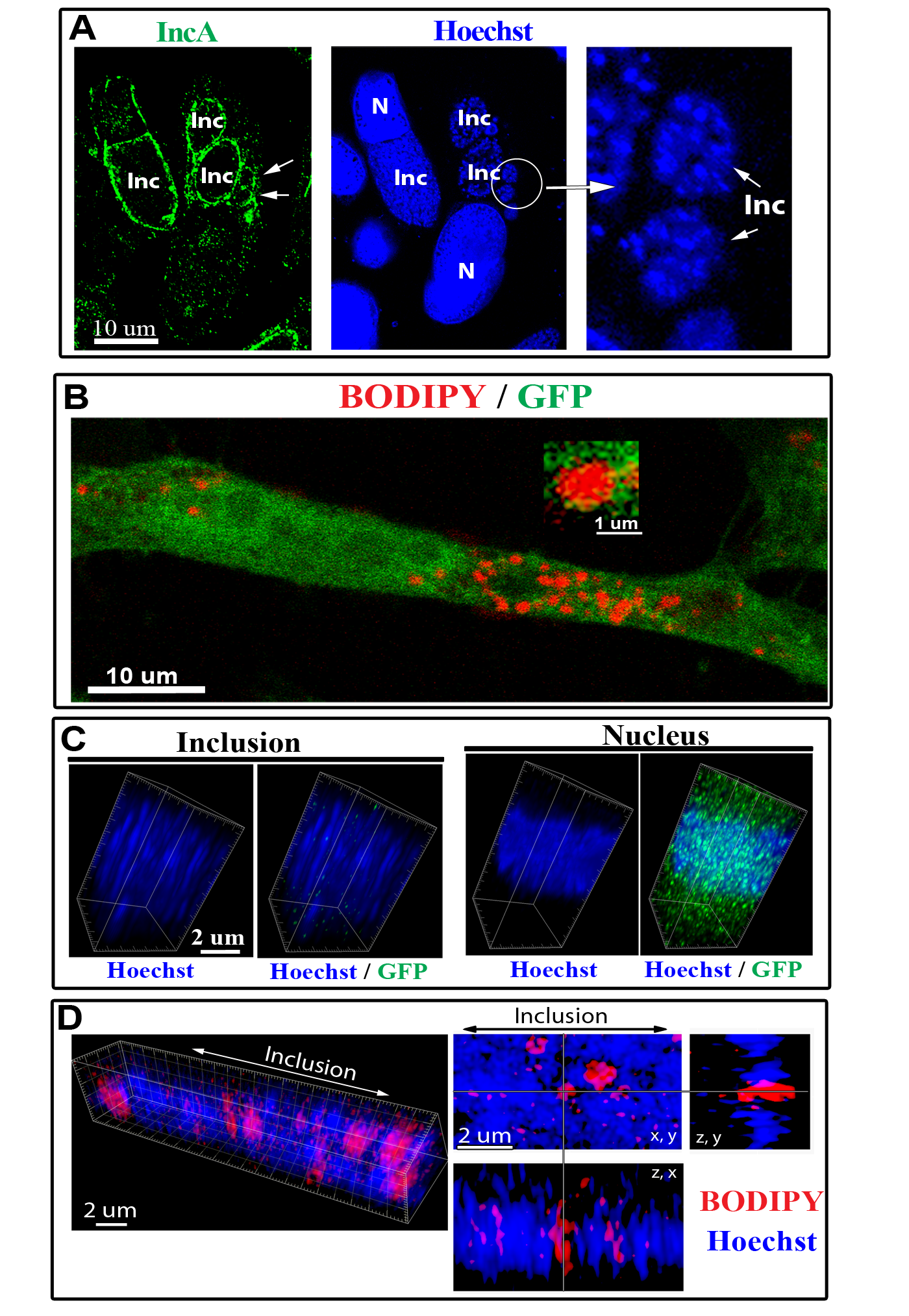

Supplement: Supplementary file 2 — Figure S2. Detection of C. trachomatis, of lipid droplets and of GFP protein. (A) This panel was generated using data previously collected and published (Soupene et al. 2012). HeLa cells infected with Chlamydia trachomatis D were fixed after 24 h infection and stained with a rabbit polyclonal antibody against the bacterial protein IncA which is located in the inclusion membrane. The image shows two infected cells. One cell has a single large inclusion and the second cell has two inclusions of medium size (indicated as Inc) and two smaller inclusions (indicated by two arrows). As presented in the result section, staining of the chromosome of Chlamydia by the Hoechst DNA dye allows detection of the inclusions as well as the nuclei. Moreover, Hoechst staining was more efficient than IncA-staining in detecting very small inclusion (right image). This methodology (detection the nuclei and Chlamydia in the blue channel, the GFP-tagged protein in the green channel and the lipid droplets in the red channel) was adopted to maximize signal intensity while avoiding signal overlap. (B) Images obtained with HeLa cells transfected with the AcGFP control vector (green) and grown in presence of oleic acid to induce production of lipid droplet (red). Inset shows the cropped view of a single LDs. GFP signal was not detected associated with LDs. (C) Images obtained with HeLa cells infected with C. trachomatis (blue) and transfected with the AcGFP control vector (green). The GFP protein encoded by this vector is detected in the nucleus of cell but it is not detected inside the inclusion. Note that this GFP protein is not translocated in the nucleus when fused to nonnuclear protein such as hLPCAT1. (D) Images obtained with HeLa cells infected with C. trachomatis (blue) and grown in presence of oleic acid to induce production of lipid droplet (red). 3D rendering of the inside of an inclusion confirming the presence of LDs is shown on the left and orthogonal x, y, z views on the right. [file mbo30004-0235-sd2.tif]

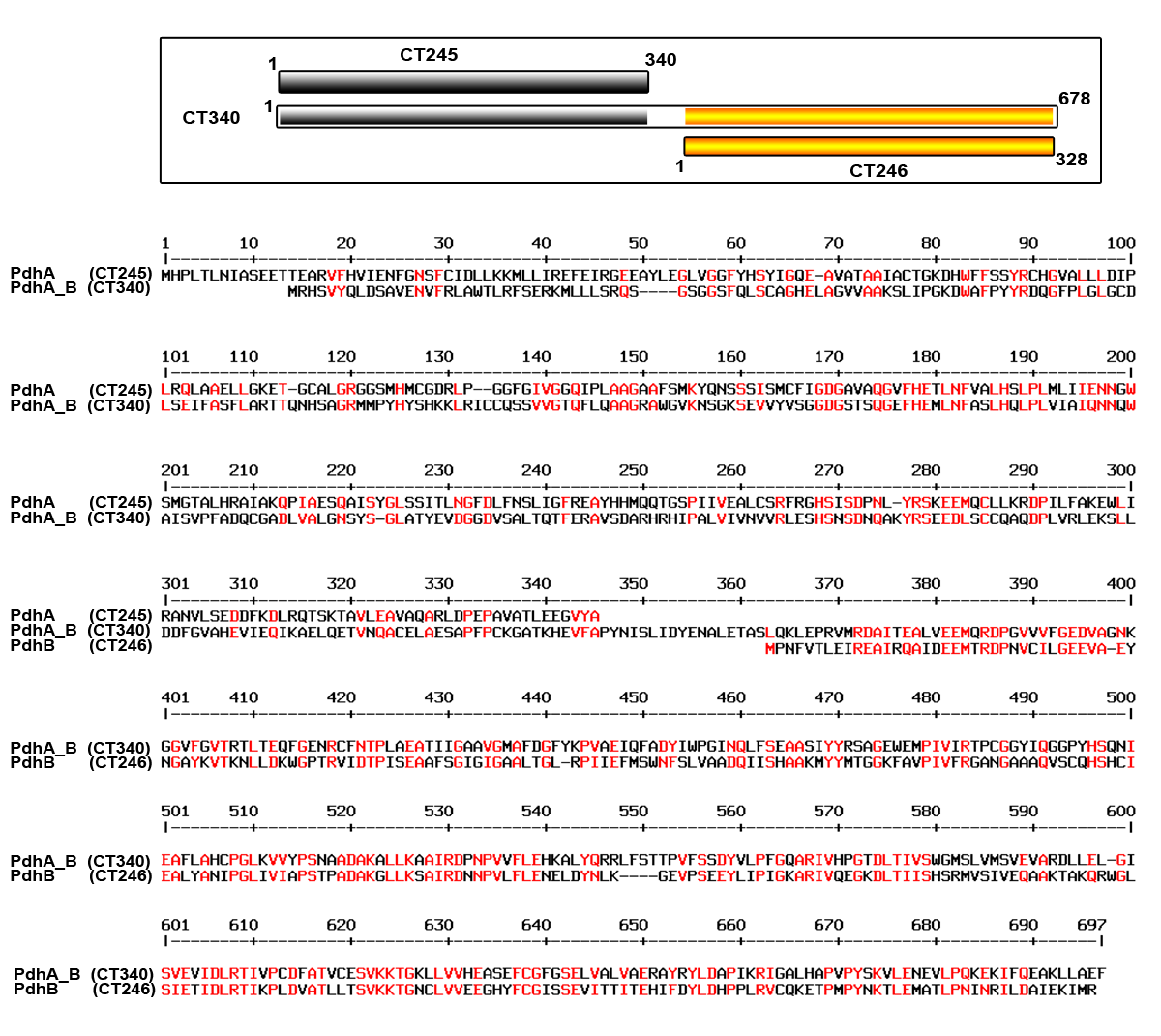

Supplement: Supplementary file 3 — Figure S3. Residues alignment of the subunits of the predicted E1 component of the PDH and BKD complexes of C. trachomatis. Alignment of the α (PdhA; CT245) and β (PdhB; CT246) subunits of the predicted E1 component of the Pyruvate Dehydrogenase (PDH) complex and of the fused α/β single subunit (PdhA_B; CT340) of the Branched αKeto Decarboxylase (BKD) complex was generated by the MultAlin program. A cartoon representation of the three subunits is shown in the inset. [file mbo30004-0235-sd3.tif]
